# Tumor Endothelial Heterogeneity in Cancer Progression

**DOI:** 10.3390/cancers11101511

**Published:** 2019-10-09

**Authors:** Nako Maishi, Dorcas A. Annan, Hiroshi Kikuchi, Yasuhiro Hida, Kyoko Hida

**Affiliations:** 1 Department of Vascular Biology and Molecular Pathology, Hokkaido University Graduate School of Dental Medicine, Sapporo 060-8586, Japan; mnako@den.hokudai.ac.jp (N.M.); annandorcasam@gmail.com (D.A.A.); 2 Vascular Biology, Frontier Research Unit, Institute for Genetic Medicine, Hokkaido University, Sapporo 060-0815, Japan; hiroshikikuchi16@yahoo.co.jp; 3 Department of Renal and Genitourinary Surgery, Hokkaido University Graduate School of Medicine, Sapporo 060-8636, Japan; 4 Department of Cardiovascular and Thoracic Surgery, Hokkaido University Faculty of Medicine, Sapporo 060-8638, Japan; yhida@med.hokudai.ac.jp

**Keywords:** tumor endothelial cell, metastasis, heterogeneity, angiocrine factor

## Abstract

Tumor blood vessels supply nutrients and oxygen to tumor cells for their growth and provide routes for them to enter circulation. Thus, angiogenesis, the formation of new blood vessels, is essential for tumor progression and metastasis. Tumor endothelial cells (TECs) that cover the inner surfaces of tumor blood vessels reportedly show phenotypes distinct from those of their normal counterparts. As examples, TECs show cytogenetic abnormalities, resistance to anticancer drugs, activated proliferation and migration, and specific gene expression patterns. TECs contain stem-like cell populations, which means that the origin of TECs is heterogeneous. In addition, since some abnormal phenotypes in TECs are induced by factors in the tumor microenvironment, such as hypoxia and tumor cell-derived factors, phenotypic diversity in TECs may be caused in part by intratumoral heterogeneity. Recent studies have identified that the interaction of tumor cells and TECs by juxtacrine and paracrine signaling contributes to tumor malignancy. Understanding TEC abnormality and heterogeneity is important for treatment of cancers. This review provides an overview of the diversity of TECs and discusses the interaction between TECs and tumor cells in the tumor microenvironment.

## 1. Introduction

Cancer is one of the leading causes of death in most of the advanced countries, and the main cause of cancer death is distant metastasis. Hematogenous metastasis is still incurable, although patient survival has improved. Understanding and overcoming tumor progression and metastasis are crucial in cancer therapy. Tumor tissues require oxygen and nutrients to grow, and these are supplied by blood flow to the tumor. Without neovascularization, most tumors may become dormant at a diameter of 2–3 mm [1]. Blood vessels support tumor cell expansion by providing the routes from intravasation in primary tumors to extravasation in distant organs. Tumor blood vessels play an important role in tumor growth and dissemination.

Antiangiogenic therapy was proposed by Dr. Folkman [1]. Since solid tumors are dependent on neovascularization for their growth, Folkman suggested that the prevention of neovascularization may restrict tumor growth to a very small diameter [1]. Angiogenic inhibitors such as bevacizumab, a humanized anti-vascular endothelial growth factor (VEGF) antibody [2], have been used for the past 15 years. Because VEGF is known as a permeability factor [3,4,5], antiangiogenic therapy not only suppresses the growth of tumors, it also normalizes blood vessel structures and improves the delivery of oxygen and drugs, which potentially affects both radiotherapy and chemotherapy [6,7]. However, the clinical benefits of antiangiogenic therapies have been limited, resulting in slight improvements in prognosis, such as enhancing progression-free survival [8]. In addition, resistance to antiangiogenic therapy has emerged because of the complex interaction between tumor cells and stromal cells, including endothelial cells (ECs), which allows for tumor cells to escape these targeted therapies [9].

Tumor endothelial cells (TECs) that cover the inner surfaces of tumor blood vessels are the primary targets of antiangiogenic therapy. Several reports have demonstrated that TECs are abnormal, and their abnormality is one of the causes of resistance to antiangiogenic therapy. In addition, TECs show intertumoral and intratumoral heterogeneity in terms of communicating with the surrounding tumor microenvironment. Reviewing how to overcome cancer from a TEC perspective, we focus on the abnormality and diversity of TECs, incorporating a discussion regarding the interaction between TECs and tumor cells in the tumor microenvironment.

## 2. Abnormalities of TECs

### 2.1. Tumor Blood Vessels and Normal Blood Vessels

At the organ level, the vasculature in the tumors from which TECs originate has an atypical morphology described as “abnormal” in terms of structure and function. Vasculature in normal nondiseased organs has an organized hierarchical structure that supports the efficient distribution of blood and its components to cells [10]. The order of blood flow in the normal vessels is from arteries to arterioles, and subsequently to capillaries, postcapillary venules, and lastly veins. In terms of function, tumor blood vessels do not support a sequential pattern of blood flow due to the chaotic order of organization.

The formation of tumor blood vessels from existing ones, called angiogenesis, occurs in response to the proangiogenic stimuli, including VEGF, basic fibroblast growth factor (bFGF), placental growth factor, and angiopoietin, among others that are produced by the tumor cells [11,12]. Hypoxia [13] and acidity [14], which are commonly associated with the tumor microenvironment, also can stimulate VEGF production in tumors. The abundance of VEGF and/or the other angiogenic factors in the tumor microenvironment sustains a continuous process of angiogenesis, leading to the formation of tumor blood vessels with various structural defects [12]. These tumor blood vessels are tortuous, highly permeable, and dilated, and show differential coverage and a loose association of perivascular cells along the vessels and weakened EC junctions [15,16].

Another important contribution to the abnormal phenotype of tumor vasculature is the insufficient control of the angiogenesis process. It has been documented that there exists an imbalance in the expression of the angiogenesis stimulators and inhibitors [17,18]. Furthermore, it was recently demonstrated that uncontrolled glycolysis in TECs due to an upregulated expression of glycolysis genes, including the enzyme 6-phosphofructo-2-kinase/fructose-2,6-biphosphatase 3 (PFKFB3), contributes to structural deformities observed in tumor blood vessels [19].

These abnormal structural changes make tumor blood vessels highly permeable compared with normal vessels. Proteins and fluids leak out of the vessels into the extracellular environment and create a high tumor interstitial pressure [20,21]. In addition, the expanding tumor population exerts more pressure on the blood vessels, causing some portions to collapse. Concomitantly, blood flow to certain portions of the tumor is cut off, leading to hypoxia [22], a switch to glycolytic metabolism in some tumors, and an increase in tumor acidosis. Hypoxia in tumors further induces tumor aggressiveness through epithelial-mesenchymal transition, resulting in tumor metastasis [23].

### 2.2. Differential Characteristics of Tumor and Normal Endothelial Cells

**Figure 1:**
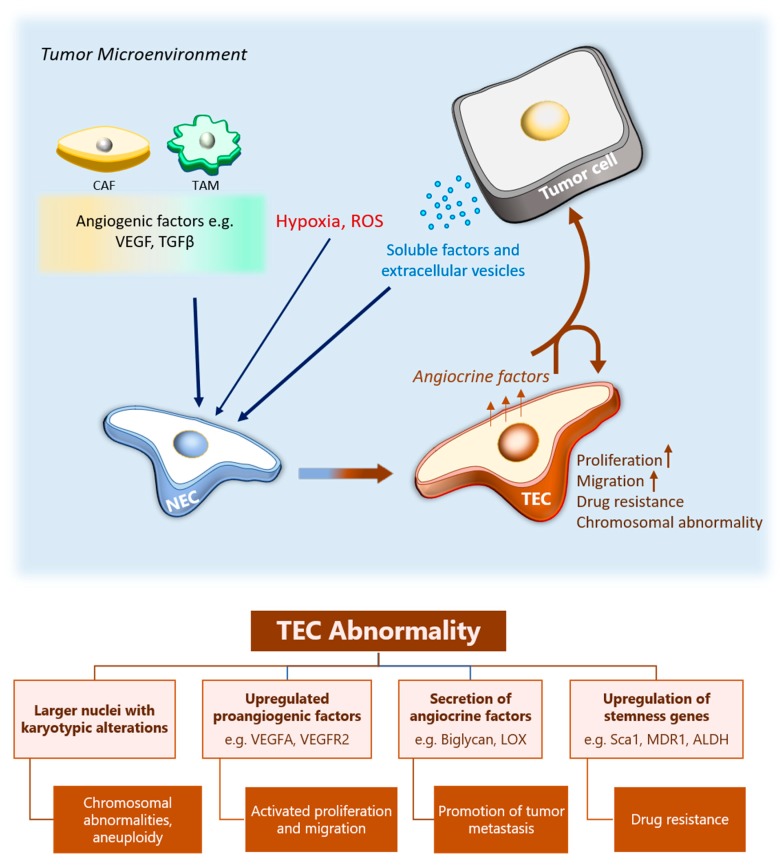
TEC abnormality. Soluble factors and extracellular vesicles released from tumor cells, CAFs and TAMs, induce endothelial cells in pre-existing blood vessels to initiate angiogenesis to form tumor blood vessels. In the process, the NECs are transformed into TECs in the formed tumor vessels. Additionally, hypoxia and ROS in the TME may contribute to the TEC phenotype. TECs have higher proliferative and migration abilities as compared to the NECs. They have an abnormal karyotype characterized by various chromosomal abnormalities and aneuploidy. The genetic changes that occur lead to the upregulated expression of proangiogenic genes e.g., VEGFA and angiocrine factors such as biglycan, which induces angiogenic function in the TECs and may also affect the tumor cells. Furthermore, the upregulation of stemness genes such as MDR1 and ALDH lead to the development of a drug resistant phenotype in the TECs. ROS, reactive oxygen species; TEC tumor endothelial cells; NEC, normal endothelial cell; CAF, cancer-associated fibroblast; TAM, tumor-associated macrophages; TME, tumor microenvironment.

Endothelial cells (ECs) in blood vessels are the primary cells in blood vessel formation, and in a similar way as tumor blood vessels show alterations compared with the normal vessels, the resident endothelial cells in tumor blood vessels are also different. Compared with normal endothelial cells (NECs), TECs differ in their genetic makeup, protein expression, and functional output (Figure 1).

We have previously reported that human renal TECs show various percentages of aneuploidy as compared to NECs [24]. Similarly, in murine TECs the karyotype indicated the presence of larger nuclei and more aneuploidy than in NECs. Within the nuclei in the TECs were chromosomal aberrations, including missing whole or portions of chromosomes, translocations, and abnormal centrosomes characterized by larger sizes and excess numbers than in NECs [25]. These observations indicate that generally TECs have chromosomal instability.

At the molecular level, TECs express angiogenesis-sustaining genes; for example, receptors such as VEGFR-1, VEGFR-2, and VEGFR-3, angiopoietin receptor tie-2, and an upregulated expression of angiopoietin 1 and VEGF-D compared with NECs [26]. With these genes, TECs exhibit a strong response to the respective angiogenic factors for the receptors [27,28]. In addition, we have also previously reported that TECs show an upregulated expression of nonconventional angiogenic factors such as biglycan [29], lysyl oxidase (LOX) [30], and pentraxin 3 (PTX3) [31]. Furthermore, TECs have been described as being “activated” and “chronically inflamed” [32]; they express adhesion molecules ICAM-1, VCAM-1, and E-selectin [27], through which they interact with proinflammatory and tumor cells.

In performing angiogenesis, both human and murine TECs are highly proliferative [28], self-sustaining, and are not dependent on serum for proliferation the way that NECs are [26]. Differentially expressed in FDCP 6 homolog (DEF6) and PTX3 play a role in regulating EC proliferation [31,33], and their expression in TECs could partly account for how TECs regulate and sustain continuous proliferation. The migration ability of TECs is also higher than that for NECs [28,34]. We have demonstrated that some genes upregulated in TECs are important for TEC migration. For example, we showed that interrupting LOX and biglycan, which were upregulated in isolated TECs, decreased migration and tube-forming ability and caused morphological changes in the TEC [29,30]. Pharmacological LOX inhibition in vivo also led to a decrease in tumor metastasis [30]. Moreover, murine TECs maintain their biological characteristics after longer periods of cell culture than do NECs [28].

The exposure of tumor cells to the hypoxic tumor microenvironment induces the expression of stemness genes [35]. Provided that TECs are exposed to a similar microenvironment, some studies have identified the upregulated expression of stemness genes such as stem cell antigen-1 (Sca-1) [28], MDR-1 [36], and ALDH [37] in the TECs. We have shown that the expression of MDR-1 [36] and ALDH in TECs that are derived from highly metastatic melanoma, for example, induces in the TECs a property of drug resistance to the drug paclitaxel [37]. Another study, involving TECs derived from a human hepatocellular carcinoma, showed that the CD105+ TECs acquired resistance to 5-fluorouracil (an anticancer drug) and sorafenib (an antiangiogenic drug) as compared to the CD105+ NECs or human umbilical vein endothelial cells (HUVECs) [38].

## 3. Heterogeneity of TECs

### 3.1. Different Roles in ECs during Angiogenesis

Angiogenesis starts in response to cues in injury or pathological condition. VEGF and other proangiogenic factors stimulate quiescent ECs and activate to adopt angiogenic phenotype. Three types of cells, namely tip, stalk, and phalanx cells, are known to coordinate the sprouting of capillaries from pre-existing vessels. Migrating tip cells lead the nascent vessel sprouts at the forefront. Proliferating stalk cells trail the tip cells and elongate blood vessels [39]. Acquiescent phalanx cells form continuous monolayers, forming a tight barrier. These specializations of ECs are transient and reversible by altering the balance between proangiogenic factors, such as VEGF, and suppressors of EC proliferation, such as Dll4-Notch activity [40,41]. Tip cells migrate in response to the VEGF gradient, while stalk cells that proliferate are dependent on the VEGF concentration [42]. Phalanx cells secrete soluble Flt1 (VEGFR-1), which neutralizes VEGF activity to end angiogenesis [43].

These ECs differ in their metabolism [44,45]. Since angiogenic sprouting is metabolically demanding [46], ECs rely on glycolysis [44], which is stimulated by the regulator PFKFB3. ECs can interchange their position depending on their metabolic condition during angiogenesis. Stalk cells overtake the tip cell position when they express higher levels of PFKFB3 [44]. This specialization is one of the heterogeneities of ECs in a tumor microenvironment.

### 3.2. Origin of TECs

Angiogenesis is the process of sprouting from a pre-existing vessel, while vasculogenesis is mediated by the mobilization of precursor cells, such as endothelial progenitor cells (EPCs) from bone marrow. EPCs were named by Asahara et al., who isolated from adult peripheral blood mononuclear cells showing the same characteristics as the embryonic angioblasts [47,48,49]. Although the identity and the contribution of EPCs in tumors are controversial and are still under discussion, several studies have shown that EPCs are incorporated into newly formed tumor blood vessels [50,51,52,53]. The surface markers of EPCs are classically expected to express CD34, VEGFR-2, and CD133 [49]. Numerous studies have aimed to target EPCs to develop novel therapeutic strategies, since EPCs or circulating endothelial precursor cells contribute to tumor angiogenesis [50,51].

ECs are heterogeneous [54]; for example, the EC structure and function are different depending on vascular size, which is described in terms of the macrovasculature, including arterial and venous, and in terms of microvascular capillaries [55]. Morphology and marker expression in ECs show differences depending on the EC origin [56,57]. Organ-specific or tissue-specific phenotypes in ECs have also been reported [55,58,59]. ECs show heterogeneity in structure and function, and in time and space [60]. TEC heterogeneity can be caused by the surrounding endothelial heterogeneity, with an activating angiogenic switch.

The concept that tumor cells could generate TECs was introduced by some groups of investigators. Streubel et al., demonstrated that chromosomal aberrations were shared by B-cell lymphoma cells and TECs, which means that TECs in B-cell lymphomas are in part tumor related [61]. In glioblastomas, some studies reported that glioblastoma stem cells may give rise to TECs [62,63,64]. Ricchi-Vitiani et al., showed that various TECs in glioblastoma carry the same genomic alteration as tumor cells, which indicates that some TECs have neoplastic origin [62]. Want et al., also demonstrated that members of a subpopulation of TECs share the same somatic mutations as glioblastoma cells, and that the stem-cell-like CD133+ fraction includes a subset of CD144-expressing cells [63]. Soda et al., demonstrated that tumor cells directly transdifferentiate into CD31+CD34+ ECs, which may play a role in resistance found toward anti-VEGF therapy [64]. On the contrary, another study showed that glioblastoma cells give rise to pericytes rather than to ECs, using lineage tracing with pericyte- or EC-specific promoter-driven fluorescent reporters [65]. The study reported that such an event wherein glioblastoma stem cells give rise to ECs may be very rare because ECs rarely carry the cancer genetic mutations, as other groups of investigators have demonstrated [65,66,67] Transdifferentiation to ECs in tumors may occur in other cell types. In multiple myeloma, tumor-derived pleiotrophin and macrophage colony-stimulating factor stimulate monocytes to induce the expression of EC markers, and the cells become transdifferentiated into ECs that incorporate into tumor blood vessels [68]. Since Fernandez et al., demonstrated that monocyte-derived immature dendritic cells behave as endothelial-like cells in the presence of specific cytokines such as VEGF [69,70], it has been proposed that dendritic cells may possibly transdifferentiate into TECs in a cytokine-rich tumor microenvironment. These variations could lead to TEC diversity.

### 3.3. Stem Cell Population in TECs

**Figure 2:**
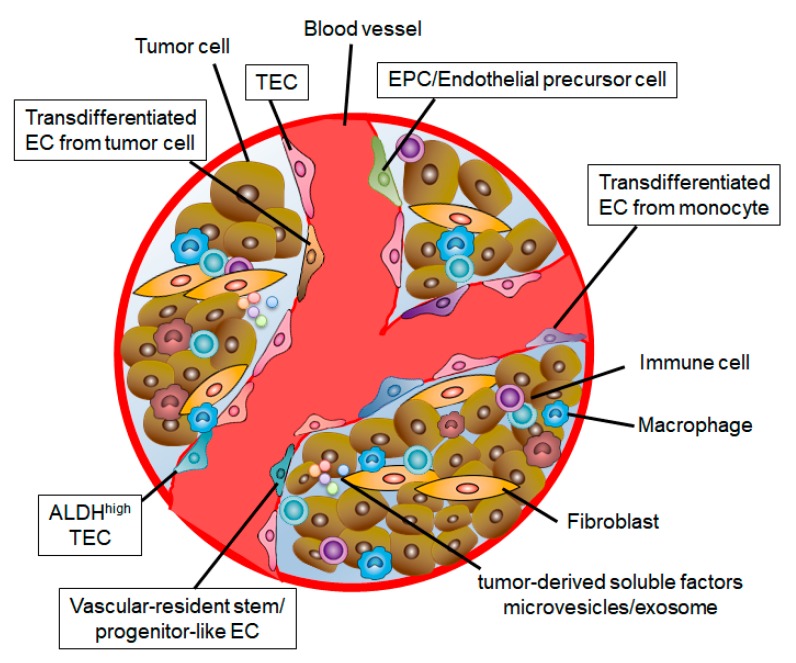
TEC heterogeneity in the tumor microenvironment. TECs are derived from multiple cells. TECs acquire their specific characteristics by several factors in the tumor microenvironment. These variations could lead to TEC diversity.

TEC heterogeneity and diversity have also been reported at functional and molecular levels [71]. TECs upregulate aldehyde dehydrogenase (ALDH) expression. There are two populations in TECs, such that some have high ALDH activity and some have low. ALDH^high^ TECs formed more tubes on Matrigel and sustained the tubular networks longer, with the upregulation of VEGFR2 expression, than ALDH^low^ TECs did [72]. The ALDH^high^ population was resistant to fluorouracil (5-FU) in vitro and in vivo, with upregulation of stem-related genes compared with ALDH^low^ TECs [37]. Naito et al., reported that vascular-resident stem/progenitor-like ECs, which form a minor population in tumors, contribute to tumor angiogenesis. Because of their ability to efflux Hoechst 33342 dye, they are termed side population cells, and cause drug resistance [73]. These reports suggested that the heterogeneity of ECs in tumor tissues may be a mechanism contributing to resistance to anticancer and antiangiogenic therapy (Figure 2).

### 3.4. The Effect of Tumor Microenvironment on TEC Heterogeneity

The heterogeneity of TECs in highly metastatic tumors and in tumors with low metastasis has been also described [34]. TECs derived from highly metastatic tumors display activated proliferation and migration with the upregulation of proangiogenic factors [34]. TECs in highly metastatic tumors show a stem-like phenotype with an upregulation of stem cell markers, such as CD90 and Sca-1, and a high ability to form spheres [34]. These data suggested that the microenvironment surrounding TECs, including tumor cell phenotype and metastatic potential, affects TEC characteristics and induces heterogeneity among TECs.

TECs acquire their specific characteristics in the tumor microenvironment during tumor angiogenesis. One of the factors in tumor microenvironment is hypoxia. It is well known that tumors are hypoxic [74]. Since the tumor blood vessel pattern is nonhierarchical and disorganized due to the excessive VEGF causing high permeability, which in turn results in an insufficient blood supply, tumor blood vessels are also sometimes exposed to hypoxia. Hypoxia induces the expression of hypoxia-inducible factor-1 alpha and transcribes several molecules, such as VEGF-A. In highly metastatic tumors, TECs are exposed to hypoxia and the expression of VEGF-A is high compared with TECs in tumors with low metastasis [34]. Hypoxia induces the accumulation of reactive oxygen species (ROS). We have previously demonstrated that ROS induces some TEC marker expression in ECs [75]. TECs show chromosomal instability [24,25], and the abnormalities are accumulated in TECs in highly metastatic tumors [34] or ALDH^high^ TECs [72]. One of the causes of cytogenic abnormality in TECs was excessive VEGF [76] and ROS [77]. These factors in the tumor microenvironment induce abnormalities and heterogeneity in ECs.

Tumor-derived soluble factors and microvesicles/exosomes are also among the causes of abnormalities in TECs. TEC marker expression was induced by tumor-derived soluble factors. For example, the expression of CXCR7 [78], biglycan [79], and MDR1 [36] was upregulated by soluble factors derived from highly metastatic tumors. Tumor-derived microvesicles induce a proangiogenic phenotype in ECs via endocytosis [80]. TECs acquire their specific characteristics by several factors in the tumor microenvironment.

Previous studies have demonstrated that endothelial-to-mesenchymal transition (EndMT) can occur in cancer [81]. EndMT is recognized as a unique source of cancer-associated fibroblasts (CAFs). These CAFs coexpress the EC marker CD31, along with one of the mesenchymal markers, FSP1, or αSMA [81]. Transforming growth factor β (TGFβ) is known to induce EndMT [82]. Conversely, recent studies demonstrated that ECs resist specific conversion to alpha-SMA+ myofibroblast-like cells when the cells are challenged with TGFβ through secretion of bFGF [83]. TGFβ and bFGF could oppose and cooperate with each other during EndMT via Elk1 [84]. These data suggest that EndMT is another mechanism producing TEC heterogeneity.

## 4. The Role of TECs in Cancer Progression

Tumors often become more malignant and aggressive progressively, step by step. In a primary tumor, tumor cells increase and gain malignant potential, and tumor cells invade the surrounding stroma and extracellular matrix (ECM). At the metastatic phase, tumor cells intravasate into vessels and reach distant organs [85]. Tumor stromal cells such as CAFs or immunosuppressive cells contribute to tumor progression [86], and TECs also play crucial roles at these steps, in addition to supplying oxygen and nutrients by blood flow. The upregulated expression of VEGF receptors may contribute to the rapid response of TECs to VEGF to facilitate disorganized blood vessel formation, through which tumor cells could get into the blood stream. Upregulation of adhesion molecules in TECs gives tumor cells scaffolds to invade between TECs, which lead to extravasation to drive metastatic dissemination [87]. In addition, TECs provide a number of inductive factors named “angiocrine factors” (Table 1), and these factors stimulate tumor growth and tumor cell migration [88]. TECs produce various molecules such as endothelin-1, bFGF, TGFβ, interleukin (IL)-6, and IL-8 as paracrine mediators of prostate cancer progression [89]. Other angiocrine factors, including IL-6, IL-3, granulocyte colony-stimulating factor (G-CSF), granulocyte-macrophage-CSF (GM-CSF), IL-1, and nitric oxide, promote leukemic cell proliferation. In addition, Jag1 derived from TECs activates Notch2 in lymphoma cells to promote tumor invasiveness [90]. CXCR7 on TECs is involved in tumor growth and angiogenesis [78,91,92]. CXCR7 regulates CXCL12-CXCR4-mediated tumor cell transendothelial migration [93]. Platelet-derived growth factor (PDGF) signaling plays a crucial role in inhibitor of differentiation 4 (ID4)-mediated regulation of ECs and Glioma-initiating cells by promoting the PDGF-NOS (nitric oxide synthase)-ID4 signaling axis. These effects maintain cancer stemness and promote tumor angiogenesis [94]. In addition, TECs downregulate tumor-suppressive factors such as Slit2. Slit2 is one of the tumor-suppressive angiocrine factors that is negatively regulated by the EphA2 receptor on ECs [95].

Moreover, TECs stimulate tumor cell intravasation and metastasis. Wieland et al., demonstrated that Notch1 in TECs activates the migration of tumor cells and promotes intravasation. Endothelial Notch1 promotes lung metastasis with neutrophil infiltration. In addition, TECs frequently express elevated Notch1 in human tumor tissues, including melanoma, breast carcinoma, lung adenocarcinoma, serous ovarian carcinoma, and colorectal carcinoma, and this expression correlates with poor prognosis [115]. ALK1 in TECs is also involved in tumor metastasis. ALK1 expression in TECs is an independent prognostic factor for metastasis of breast cancer [116]. The oxygen-sensing prolyl hydroxylase domain protein 2 (PHD2) in TECs is involved in vessel shaping. Haplodeficiency of PHD2 did not affect vessel density or lumen size, however, it normalized the endothelial lining and vessel maturation in tumors, which leads to the reduction of tumor cell intravasation and metastasis [43]. We have shown that biglycan, a small leucine-rich repeat proteoglycan, was remarkably upregulated in TECs and facilitated the migration of toll-like receptor-expressing tumor cells, which increased circulating tumor cells and lung metastasis [79]. Endothelial calcineurin have a unique function, which does not affect primary tumor growth, but activates the outgrowth of metastases [99]. These studies suggested that TECs actively promote tumor cell progression and metastasis.

Drugs for anticancer treatment include cytotoxic drugs and molecular targeting drugs. In most cases, these drugs gradually become ineffective in cancer treatment, and this is considered to be caused by tumor cells acquiring drug resistance. It is generally known that tumor cells acquire drug resistance via phenotypic changes, such as increased drug transporter expression [117]. On the contrary, TEC characteristics also cause drug resistance. Renal cell carcinoma-derived TECs are resistant to vincristine [26], and hepatocellular carcinoma-derived TECs are resistant to 5-FU and adriamycin [36,38]. TEC-derived Jag1 confers Notch-dependent chemoresistance in lymphoma cells [90]. TECs have acquired resistance to anticancer drugs via the upregulated expression of ATP-binding cassette transporters, similar to cancer stem cells [36,118]. TECs also play a role as a molecular checkpoint in chemotherapy. IGFBP7 expressed by TECs suppresses IGF1R signaling and the stem-cell-like property of tumor cells. Chemotherapy triggers TECs to suppress IGFBP7, and the upregulation of IGF1 activates the FGF4-FGFR1-ETS2 pathway in TECs and accelerates the conversion of tumor cells to chemoresistant tumor stem-like cells [105]. The drug resistance property of TECs also serves to promote tumor survival. Residual TECs in drug-treated tumors will restore angiogenesis in the more resistant tumor cells that have survived antiangiogenic therapy.

In recent years, tumor immunity has been noted as an important factor for anticancer treatment, and immune checkpoint inhibitors have become key drugs for antitumor immunity [119,120]. ECs play an important role in controlling immune cell entry into tissues with chemokines and adhesion molecules [121]. In tumor tissues, the abnormalities of TECs suppress T-cell trafficking and function and cause an immune-suppressive environment [122]. For example, the high expression of VEGF and other growth factors reduces endothelial ICAM-1 and VCAM-1 expressions in tumor tissues, causing the lymphocyte–endothelial interactions to become inefficient [123]. VEGF and prostaglandins induce CD95 (FasL) expression on TECs, leading to apoptosis of activated anticancer CD8+ T cells. In contrast, regulatory T cells that suppress antitumor immune responses are protected by antiapoptotic genes, such as Bcl2 and Bclxl, and these cells can selectively migrate into tumor tissues [124]. Upregulation of CD73 on TECs reduces effector T-cell homing, whereas anti-CD73 antibodies can restore efficacy of antitumor immunotherapy and decrease tumor angiogenesis [125,126]. In addition, PD-L1, which is a negative regulator of T-cell activation, is expressed in TECs. PD-L1 blockade enhances tumor vascular normalization during anti-VEGF therapy [127]. Macrophage also plays an important role for tumor immunity. TECs are one of the major sources of IL-6 in glioblastoma. Angiocrine IL-6 induces arginase-1 expression and promotes macrophage alternative activation [107]. Thus, vascular normalization is a promising concept in anticancer treatment and can potentially improve the outcome of immunotherapies [128,129,130].

## 5. Conclusions

In this review, we addressed the abnormality and heterogeneity of TECs to understand their roles in the tumor microenvironment. The functions of ECs in newly formed blood vessels in tumor tissues are not only to transport nutrients and oxygen for tumor survival and growth, but also to actively promote tumor progression and chemoresistance.

Antiangiogenic therapy has been widely used in many types of tumors; however, since it is now clear that TECs are heterogeneous, to overcome and regulate tumor angiogenesis is a difficult and urgent task. To understand the complex situation in the tumor microenvironment, companion diagnostics to monitor vascularization is required. In addition, both angiogenesis and vasculogenesis need to be targeted to regulate aberrant excessive blood vessels. Targeting multiple growth factors as combination therapy have shown improved outcomes, but the therapeutic effects are sometimes not enough. Another therapy, such as combination with immunotherapy or targeting EC metabolism is expected to normalize tumor microenvironment to cure cancer disease.

## Figures and Tables

**Table 1:** Angiocrine factors produced by tumor endothelial cells.

Angiocrine Factors	Functions	Refs
Angiopoietin-2 (Ang2)	Recruit innate immune cells	[96]
Basic fibroblast growth factor (bFGF)	Organogenesis and tumorigenesis	[89,97]
Biglycan	Stimulate tumor cell intravasation	[79]
Bone morphogenetic protein-2, 4 (BMP2, 4)	Tumorigenesis	[98]
Calcineurin	Vascular stabilization and promote metastatic outgrowth	[99]
C-X-C motif chemokine 12 (CXCL12)	Tumorigenesis and tumor progression	[100,101]
Endothelin-1	Promote tumor growth	[102]
Granulocyte colony stimulating factor (G-CSF)	Promote leukemic cell proliferation	[103]
Granulocyte macrophage colony stimulating factor (GM-CSF)	Angiogenesis	[104]
Insulin growth factor binding protein-7 (IGFBP7)	Tumor-suppressive checkpoint	[105]
Insulin growth factor-1 (IGF1)	Stimulate chemoresistance and angiogenesis	[105,106]
Interleukin-3 (IL-3)	Promote leukemic cell proliferation	[103]
Interleukin-6 (IL-6)	Macrophage activation and tumor progression	[107]
Interleukin-8 (IL-8)	Angiogenesis and tumor progression	[108]
Jagged-1 (Jag1)	Promote tumor invasiveness and chemoresistance	[90]
laminin α4 (LAMA4)	Tumorigenesis	[109]
Lysyl oxidase (Lox)	Angiogenesis and stimulate tumor cell intravasation	[30]
Nitric oxide (NO)	Tumorigenesis	[110]
Platelet-derived growth factor (PDGF)	Angiogenesis and tumorigenesis	[94]
Placental growth factor (PGF)	Angiogenesis and tumorigenesis	[111]
Pentraxin 3 (PTX3)	Stimulate TEC proliferation	[31]
Slit2	Tumor suppression	[95]
Suprabasin	Angiogenesis	[112]
Transforming growth factor beta (TGF-β)	Tumorigenesis and tumor progression	[113]
Vascular endothelial growth factor-A (VEGFA)	Angiogenesis and autocrine loop	[114]
